# Gut bacterial extracellular vesicles: important players in regulating intestinal microenvironment

**DOI:** 10.1080/19490976.2022.2134689

**Published:** 2022-10-15

**Authors:** Xiao Liang, Nini Dai, Kangliang Sheng, Hengqian Lu, Jingmin Wang, Liping Chen, Yongzhong Wang

**Affiliations:** a School of Life Sciences, Anhui University, Hefei, China; b Key Laboratory of Human Microenvironment and Precision Medicine of Anhui Higher Education Institutes, Anhui University, Hefei, China; c Anhui Key Laboratory of Modern Biomanufacturing, Anhui University, Hefei, China; d Institute of Physical Science and Information Technology, Anhui University, Hefei, China

**Keywords:** Bacteria-bacteria interaction, bacteria-host interaction, gut bacterial extracellular vesicles, intestinal dysbiosis, intestinal microenvironment

## Abstract

Intestinal microenvironment dysbiosis is one of the major causes of diseases, such as obesity, diabetes, inflammatory bowel disease, and colon cancer. Microbiota-based strategies have excellent clinical potential in the treatment of repetitive and refractory diseases; however, the underlying regulatory mechanisms remain elusive. Identification of the internal regulatory mechanism of the gut microbiome and the interaction mechanisms involving bacteria-host is essential to achieve precise control of the gut microbiome and obtain effective clinical data. Gut bacteria-derived extracellular vesicles (GBEVs) are lipid bilayer nanoparticles secreted by the gut microbiota and are considered key players in bacteria-bacteria and bacteria-host communication. This review focusses on the role of GBEVs in gut microbiota interactions and bacteria-host communication, and the potential clinical applications of GBEVs.

## Introduction

The intestinal microenvironment is the most important and complex micro-ecological host system, maintained by the microbial community and the host immune system. In addition to microorganisms, such as bacteria, archaea, protists, fungi, and viruses and their metabolites, components of the intestinal microenvironment include intestinal secretions, intestinal epithelial cells (IECs), and cytokines released during host immune responses.^1,2^ The intestinal microenvironment plays key roles in nutrient digestion, absorption, and metabolism in the intestine, development of organs, such as gut and brain, and synergistically regulating the host immune system.^3^ Dysbiosis of the intestinal microenvironment is closely associated with the initiation and progression of diseases such as common digestive system diseases,^4^ inflammatory bowel disease (IBD), ^5^ functional gastrointestinal disorders,^6^ and chronic gastritis.^7^ Additionally, it is also associated with distal organ lesions, such as those observed in cancer,^8^ and diseases of the cardiovascular system,^9^ brain,^10^ kidney,^11^ liver,^12^ and pancreas.^13^ Intestinal microenvironment dysbiosis mainly manifests as a change in the proportion or number of colonization sites of intestinal microbes. Particularly, an imbalance of the intestinal microbiota occurs, resulting in a loss of host functions including energy metabolism, signal transduction, pathogen resistance, and immune regulation, and finally inducing or worsening a disease. Such changes are mainly attributed to a decrease in the abundance and diversity of commensal microflora coupled with an increase in the abundance of pathogenic bacteria. In a nutshell, the intestinal microenvironment is inextricably associated with the optimal functioning of the gut. Effective intervention strategies to restore homeostasis of the intestinal microenvironment include fecal microbiota transplantation (FMT) and the use of microecologics.^14,15^

The gut microbiota is predominantly composed of bacteria. More than 1000 bacterial species are commonly found in the intestinal tract.^1^ The composition of the gut microbiota is influenced by host factors such as intestinal environment, diet, and age.^16–18^ On the other hand, the physiological and psychological states of the host are influenced by gut bacterial communities.^19,20^ Studies in mice have demonstrated that up to 70% of the intestinal chemicals are regulated by gut bacterial communities. Moreover, such chemical regulation is observed in organs distant from the gut, such as uterus or brain, 20% of the compounds are regulated by gut bacteria.^21^

**Figure 1.**
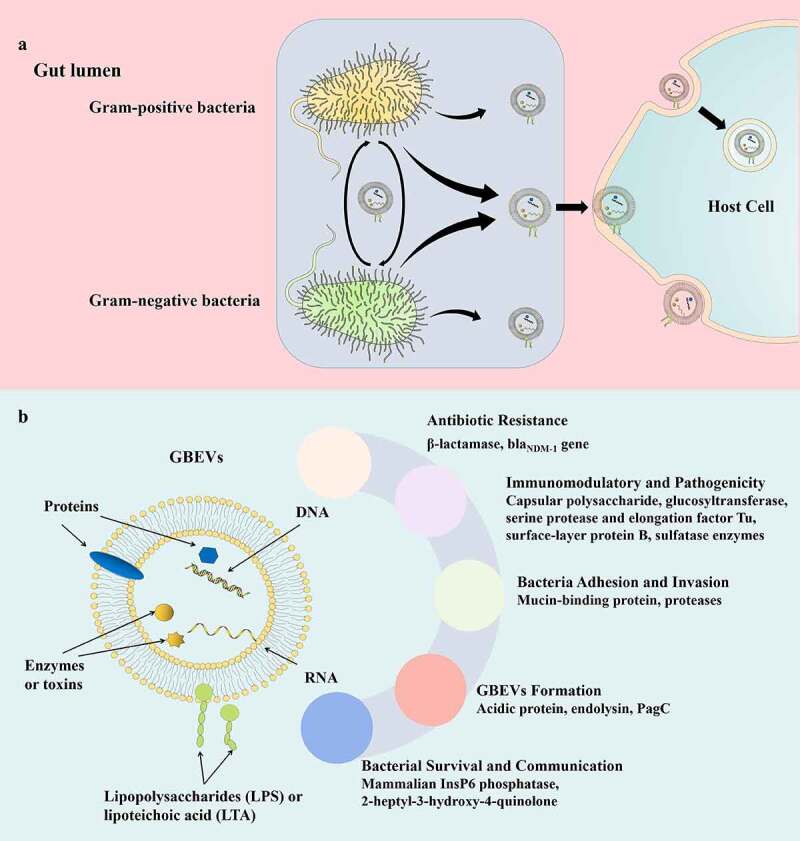
(a) Proposed critical pathophysiological functions of GBEV cargos. (b) Schematic representation of bacteria-host and bacteria-bacteria GBEVs interactions. GBEVs: gut bacteria-derived extracellular vesicles.

Although gut bacteria are critical for the regulation of the intestinal microenvironment and host health, the mechanisms underlying this regulation have been largely unexplored.^22,23^ However, recently, bacterial extracellular vesicles (BEVs) secreted by intestinal commensal bacteria, probiotics, and pathogenic bacteria were identified to modulate intestinal microenvironment and host health.^24–26^ Extracellular vesicles (EVs) are defined as nano-vesicles that are secreted by cell membranes of live cellular organisms such as eukaryotes, bacteria, archaea, and mycoplasma. EVs are mostly spherical in shape and 20–400 nm in diameter.^27^ The vesicle membrane is a lipid bilayer and may encapsulate molecules such as DNA, RNA, proteins, peptidoglycan, and lipids (Figure 1A).^28,29^ The development of omics technology has helped elucidate the chemical composition of EVs. However, most of the current studies on cargo mainly focus on structural and functional analysis of protein components, with few studies considering other EV components such as lipids, nucleic acids, and polysaccharides.

In 1981, Wensink and Witholt speculated that excessive production of the bacterial outer membrane was the leading cause of BEV generation;^30^ however, this hypothesis failed to explain the formation of BEVs under normal growth conditions. In 1998, Leah et al. proposed that gram-negative BEVs result from cell wall turnover, and peptidoglycan turnover causes the outer membrane to bulge and finally bleb, followed by mechanical motion of the cell wall to release BEVs.^31^ Although this was demonstrated in the formation of *Porphyromonas gingivalis* EVs, further research revealed that the biogenesis of BEVs is a complex process, and various factors affect their secretion. So far, a consensus mechanism underlying BEV biogenesis applicable to all bacteria has not been identified.^32,33^ BEVs secreted by gram-negative bacteria are also referred to as outer membrane vesicles (OMVs).^34^ Gut bacteria-derived extracellular vesicles (GBEVs) were first identified in the electron microscopic images of *Escherichia coli* by Work et al. in 1966,^35^ and a similar structure was found in sections of *Vibrio cholerae* by Chatterjee et al. in 1967.^36^ The concept of BEVs was explicitly proposed by Dorward et al. in 1990.^37^ Subsequently, research surrounding gram-positive bacteria, gram-negative bacteria, and archaea in the intestinal tract confirmed the presence of GBEVs.^28^ Since then, the structure, biogenesis, and interactions within the intestinal flora and with the host of GBEVs has attracted tremendous research interest. Regulation of GBEVs in the intestinal microenvironment and host health has also become an emerging research field in the study of intestinal microbial interactions and bacteria-host interactions (Figure 1B). A variety of commensal bacteria as well as enteric pathogens are present in different stages of the gut (in diseased states or physiological metabolic disorders). To better illustrate the role of GBEVs in the intestinal microenvironment, in addition to GBEVs secreted by intestinal commensal bacteria, GBEVs secreted by enteric pathogens are included in this review. Herein, we describe the regulatory role of GBEVs in the intestinal microenvironment played by bacteria-bacteria and bacteria-host communication, and discuss their possible clinical implications.

## GBEVs-mediated bacteria-bacteria interactions

Currently, the regulation of gut microbiota, such as through FMT and probiotic intervention, to restore homeostasis of the intestinal microenvironment is an important therapeutic strategy for recurrent and refractory diseases^38^ and exhibit excellent clinical therapeutic effects. Nonetheless, the mechanisms underlying these strategies are still unclear. Intestinal bacteria can communicate with the surrounding environment by secreting chemicals such as toxins, quorum sensing (QS) molecules, and nucleic acids. GBEVs, as carriers of these signaling molecules, play an important role in regulating the *in vivo* balance of intestinal bacteria (summarized in Table 1).

**Table 1.** The roles of GBEVs in communication cross-talk between gut bacteria.

GBEVs source	Cargos of GBEVs	Function of GBEVs	Refs
*Bacteroides fragilis*	Acidic lipoproteins	Degradation of various polysaccharides.	^39^
*Bacteroides thetaiotaomicron*	1. β-lactamases; 2. Acidic lipoproteins	1. Protect commensal bacteria and enteric pathogens against β-lactam antibiotics; 2. Degradation of various polysaccharides.	^39,40^
*Bifidobacterium longum*	Mucin-binding proteins	Promote bifidobacterial colonization.	^24^
*Escherichia coli*	Unknown	Protection of parental bacteria by adsorption of colistin.	^41^
*Escherichia coli* O104:H4	*bla_CTX-M-15_*	Transmission of antibiotic resistance gene.	^42^
*Pseudomonas aeruginosa*	2-heptyl-3-hydroxy- 4-quinolone (PQS)	1. Communicate and coordinate social activities; 2. Promotes biofilm diffusion; 3. Aids in the absorption of iron molecules by bacteria.	^43–45^
*Vibrio cholerae* C6706	PrtV protease	Helps bacteria resist host defense peptides.	^46^
*Vibrio cholerae* V:5/04 non-O1 non-O139 clinical isolate	Unknown	Inhibit innate immune response of host and promoting bacterial colonization.	^47^

GBEVs: gut bacteria-derived extracellular vesicles; PQS: *Pseudomonas* quinolone signal; Refs: references.

### GBEVs promote bacterial colonization and growth

In the intestinal microenvironment, GBEVs can both help the colonization of intestinal bacteria and promote their proliferation. As an example, GBEVs are loaded with adhesion factors that promote intestinal bacterial colonization and carry polysaccharide-degrading enzymes which breakdown complex polysaccharides and provide a source of carbohydrates for the intestinal bacteria.^24,48^

Bacterial surface adhesion molecules, such as surface-exposed human plasminogen receptor,^49^ enolase,^50^ sialidase,^51^ fimbriae,^52^ and transaldolase,^53^ are critical for mediating bacteria-host interactions. Recently, Nishiyama et al. identified that the GBEVs secreted by *Bifidobacterium longum* NCC2705 play a key role in the colonization process.^24^ Biomolecular interaction analysis has revealed that GBEVs contain a large number of cytoplasmic proteins that bind to porcine gastric mucin such as chaperone GroEL, elongation factor Tu, phosphoglycerate kinase, Tal, and heat shock protein 20. In addition, these proteins were recombinantly expressed in *E. coli*, immobilized on microbeads, and were found to adhere to the gastrointestinal tract of mice.^24^ These findings suggest that GBEVs can effectively assist the colonization of *B. longum* by transporting bacterial adhesion factors into the extracellular environment.

GBEVs may also promote enteric pathogen colonization by reducing the host innate immune response. Bitar et al. coincubated *V. cholerae* V:5/04 non-O1 non-O139 clinical isolate (a highly motile gram-negative bacterium that colonizes the small intestine and causes cholera) secreted GBEVs with T84 cells for 2 h.^47^ The expression of microRNAs was determined using quantitative real-time polymerase chain reaction, and a significant increase in miR-146a expression level was observed. Elevated expression levels of miR-146a inhibit the host’s innate immune response. Interestingly, no changes in mRNA expression levels of pro-inflammatory factors, including interleukin (IL)-8, tumor necrosis factor alpha (TNF-α), and IL-1β, were observed. This effect prevents *V. cholerae* from being immediately cleared by the host immune system, thereby allowing *V. cholerae* to colonize the host gut.^47^ Although research exploring the role of GBEVs in colonization of intestinal bacterial is limited, GBEVs are presumed to exert some specific advantages in bacterial colonization because they play an important role in host-bacteria communication. In the future, GBEVs may play a central role in strategies aimed at regulating the host gut bacterial ecological balance. For instance, GBEVs could be used to enhance probiotic colonization, which is critical for the therapeutic effect of probiotics, or GBEVs could be disrupted to reduce the colonization of pathogenic bacteria.

GBEVs can provide nutrients to other bacteria by delivering a variety of enzymes involved in the degradation of complex polysaccharides. Valguarnera et al. analyzed the protein composition of *Bacteroides thetaiotaomicron* EVs using mass spectrometry and identified several glycosidases and proteases, which were mainly encoded by the polysaccharide utilization loci. When carried by GBEVs and released into the intestinal lumen, enzymes break down polysaccharides present in ingested food or host-synthesized sugar adducts into mono- or oligo-saccharides, thus promoting the growth of other bacteria.^48^ Researchers further investigated the envelope mechanism and found that it is mediated by a lipoprotein export sequence (LES, a negatively charged rich amino acid motif). Alpha-amylase starch utilization system (Sus) G is mediated by LES from the periplasmic surface of outer membranes to the extracellular environment, which in turn is preferentially encapsulated by GBEVs. On co-culturing SusG-containing GBEVs with the Δ*SusG* strain (*SusG* gene knockout strain, which cannot grow in media containing starch as the sole carbon source), the growth of the Δ*SusG* strain was found to be similar to that of the wild-type.^48^ Therefore, uncontrolled intestinal flora can be regulated by GBEVs carrying specific effectors, and thus effectively balance the host intestinal microenvironment. In addition to *B. thetaiotaomicron* EVs, polysaccharide-degrading enzymes were also detected in *Bacteroides fragilis* EVs, indicating that the encapsulation behavior is not specific to *B. thetaiotaomicron*.^39^

### GBEVs protect bacteria from antibiotics and host defense peptides

**Figure 2.**
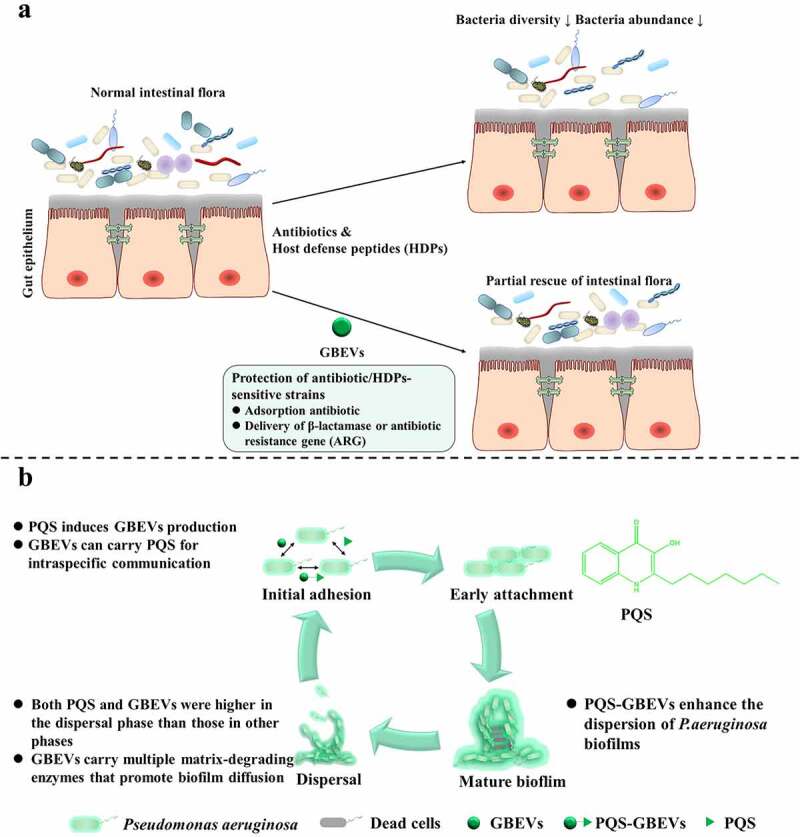
GBEVs mediate cross-talk between gut bacteria. (a) GBEVs can protect strains sensitive to antibiotics/HDPs. Treatment with antibiotics or HDPs disrupts the normal intestinal microbiota, leading to a reduction in both the diversity and abundance of intestinal bacteria, resulting in dysbiosis. GBEVs, through direct antibiotic adsorption^41^ and intra- and interspecies delivery of β-lactamase^40^ or antibiotic resistance gene^42^ can partially recovery. (b) PQS-GBEVs are involved in the developmental cycle of *Pseudomonas aeruginosa* biofilms.^43^ PQS can promote the secretion of GBEVs in *P. aeruginosa*.^54^ Moreover, PQS can coordinate population behavior through intra-species communication via GBEVs.^45^ GBEVs: gut bacteria-derived extracellular vesicles; PQS, *Pseudomonas* quinolone signal; PQS-GBEVs: PQS containing GBEVs.

The greatest threat to the existence of host intestinal bacteria are antibiotics and host defense peptides (HDPs). Consequently, bacteria have evolved various effective defense strategies against antibiotics and HDPs, and GBEVs play vital roles in executing these strategies. GBEVs produce a protective effect by adsorbing antibiotics. For example, to protect antibiotic/HDPs-sensitive strains GBEVs adsorb antibiotics and deliver β-lactamase or antibiotic resistance gene (ARG) (Figure 2A).

Colistin is an amphiphilic polypeptide antibiotic that exerts bactericidal or antibacterial effect by electrostatically binding to lipopolysaccharide (LPS) on the outer membrane of gram-negative bacteria, causing Ca^2+^ and Mg^2+^ to move out of the phosphate group of LPS, resulting in disruption of the bacterial outer membrane and efflux of cell contents.^55^ The main cause of colistin resistance in gram-negative bacteria is the presence of mobilized colistin resistance (mcr) gene (*mcr-1* to *mcr-10*).^56^ For example, *mcr-1* gene expression results in phosphoethanolamine (pEtN) modification of phospholipid A on the outer membrane of *E. coli*, which reduces the negative charge of LPS and lowers its affinity for colistin.^56^ Interestingly, GBEVs secreted by *E. coli* with positive and negative *mcr-1* expression showed differences in protective effect from parental bacteria. You and Zhang’s team found that both *mcr-1* positive or negative *E. coli* EVs protected colistin-sensitive *E. coli* strains from the effects of colistin.^41^ They also found that GBEVs secreted by *mcr-1*-negative *E. coli* provide better protection than those secreted by *mcr-1*-positive *E. coli*. The main reason for this phenomenon is that the protective effect developed by *E. coli* EVs is primarily due to their adsorption to colistin. The lipid A on the surface of GBEVs secreted by *mcr-1*-positive *E. coli* is the same as that of the parental bacteria and is also modified by pEtN, leading to a weakened binding of *mcr-1*-positive *E. coli* EVs to colistin, ultimately reducing the protective effect.^41^

In addition to antibiotic adsorption to protect sensitive strains, GBEVs can serve as a delivery platform for dissemination of antibiotic-degrading enzymes and ARGs. Among gut bacteria, anaerobic *Bacteroides* spp. are the most resistant to penicillins and cephalosporins.^57^ *Bacteroides* degrade penicillin by secreting β-lactamase^58^ or excrete antibiotics through Resistance-Nodulation-Division and multidrug and toxic compound extrusion efflux pumps.^57^ Stentz et al. determined that GBEVs secreted by *B. thetaiotaomicron* carry β-lactamases that degrade cefotaxime.^40^ Following proteinase K treatment, 84% of the β-lactamase enzymatic activity was lost, indicating that most of the β-lactamase contained in *B. thetaiotaomicron* EVs was located on the GBEVs surface. The team further studied GBEVs secreted by other bacteria of the genus *Bacteroides* (*B. fragilis* and *B. stercoris*) and detected high degradation activity for cefotaxime, thus protecting themselves from antibiotics. Furthermore, after adding *B. thetaiotaomicron* EVs to the media of *Salmonella* Typhimurium and *Bifidobacterium breve*, both grew normally in 1 mg/L of cefotaxime or ampicillin. Thus, GBEVs loaded with β-lactamase protect the parental as well as other bacteria from antibiotics.^40^ A homologous system was also observed in pathogenic *E. coli* ST131.^59^ Horizontal gene transfer (HGT) is a unique mechanism by which bacteria rapidly evolve and adapt to complex environments. In addition to transformation, transduction, and conjugation, GBEVs are often used as a fourth mode of HGT among bacteria.^60^ Bielaszewska et al. found that the diarrhea-causing enteric pathogen *E. coli* O104:H4 can deliver multiple ARGs (such as *bla_CTX-M-15_* and *bla_TEM-1_*) to other bacteria *via* GBEVs.^42^ The investigators determined that, compared to that of the untreated group, the transfer frequency of *bla_CTX-M-15_* in simulated intraintestinal environment (simulated ileal environment and colonic environment medium) and under the stress of ciprofloxacin (a drug commonly used to treat diarrhea caused by bacterial pathogens) increased approximately 100-fold.^42^ This transfer behavior is extremely beneficial for the intestinal bacteria and protects them from antibiotics; however, it possibly poses a major problem for patients with bacterial infections.

Apart from antibiotics, the most frequently encountered antimicrobial agents by gut bacteria *in vivo* are HDPs. HDPs are short peptides that exert antimicrobial effects primarily by interacting with bacterial membranes, causing membrane cleavage.^61^ HDPs are often used as alternatives to antibiotics because they can target multiple bacterial sites to exert antibacterial effects, making it difficult for bacteria to develop resistance. Veldhuizen’s team added CATH-2 (chicken-derived cathelicidin),^62^ PMAP-36 (pig-derived cathelicidin),^63^ and LL-37 (the only human cathelicidin)^64^ to *E. coli* medium and found that *E. coli* EVs effectively protected the bacteria from HDPs, and this protection was positively correlated with the amount of GBEVs.^65^ In addition, CATH-2 and PMAP-36 would further stimulate *E. coli* to secrete more GBEVs and enhance its defense against HDPs. Fortunately for the host, although LL-37 was not effective as a bactericide under the protection of GBEVs, the administration of LL-37 also did not induce the production of more GBEVs thereby enhancing the protective effect on the bacteria. Additionally, the GBEVs secreted by *E. coli* induced with 2.5 mM CATH-2 (2.5C GBEVs) not only failed to protect *E. coli*, but even enhanced the bactericidal effect of LL-37. This may be due to the continued presence of active CATH-2 in 2.5C GBEVs, which can exert a synergistic antibacterial effect with LL-37.^65^ GBEVs from *V. cholerae* C6706 can also protect parental bacteria from HDPs. Rompikuntal et al. added sublethal concentrations (25 μg/mL) of LL-37 to Luria Bertani medium containing *V. cholerae* C6706. The treated group, without the addition of *V. cholerae* C6706 EVs, exhibited significant growth lag compared with that of the control group, whereas the addition of *V. cholerae* C6706 EVs ameliorated the inhibitory effect of LL-37 on *V. cholerae* growth.^46^ Here, the protective effect was attributed to the PrtV protease loaded in GBEVs (PrtV-GBEVs), which protected the bacteria by degrading LL-37. However, replacement of PrtV-GBEVs with GBEVs secreted by the Δ*prtV* mutant strain was also protective, although the effect was weaker than that with the PrtV overexpression strain and the wild-type strain. Thus, the protective mechanism may not only rely solely on the PrtV protease, but may also be due to the adsorption of some HDPs by GBEVs.

In summary, some GBEVs can effectively help the defend the gut bacteria against antibiotics and HDPs and significantly improve their survival in the host. However, bacterial resistance is a crucial concern and is a major challenge for the global public health system. A comprehensive understanding of how GBEVs influence the interactions of gut bacteria with antibiotics and HDPs will help facilitate the clinical applicability of GBEVs. Furthermore, although only ARG transfer by intestinal bacteria through GBEVs has been reported, HGT *via* GBEVs is undoubtedly an effective strategy for the evolution of intestinal bacteria and resistance to external environmental pressures. For example, using engineered GBEVs to deliver exogenous genes of interest to dysfunctional host intestinal flora, thereby exerting regulatory effects may be another effective strategy to regulate intestinal microenvironment homeostasis using GBEVs.

### GBEVs deliver QS molecules to coordinate bacterial population behavior

QS molecules are signaling substances used by bacteria to coordinate community behavior, such as regulating bacterial symbiosis and competition, influencing the secretion of bacterial metabolites and virulence factors, and regulating biofilm formation(Figure 2B).^66^ Currently, few studies have investigated the delivery of QS molecules by GBEVs. Of those studies, most have focused on the GBEVs secreted by the opportunistic pathogen *Pseudomonas aeruginosa* and the production of 2-heptyl-3-hydroxy-4-quinolone (*Pseudomonas* quinolone signal, PQS). These studies have suggested that the interaction between PQS and *P. aeruginosa* EVs can be considered “synergistic”. Cooke et al. found that most wild-type *P. aeruginosa* strains require induction by PQS to secrete GBEVs.^67^ Further, Bala et al. observed that GBEV production was significantly reduced in the *pqs* gene mutants of *P. aeruginosa*, and that exogenous supplementation with PQS induced a significant release of GBEVs by this strain again.^54^ The mechanism underlying PQS-containing GBEV (PQS-GBEV) production in *P. aeruginosa* broadly involves the insertion of PQS into the bacterial outer membrane, which leads to the expansion of the outer leaflet, resulting in the bending of the outer membrane and the eventual formation of PQS-GBEVs.^68^ Subsequently, PQS can enter other *P. aeruginosa* cells *via* GBEVs, thereby regulating the behavior of the *P. aeruginosa* population.

PQS-GBEVs can be used to regulate the behavior of *P. aeruginosa*. Bacterial biofilms are a major contributor to chronic infections in humans (>65%)^69^ and antibiotic-resistant biofilms formed by *P. aeruginosa* can cause persistent infections at surgical sites and burn wounds.^70^ PQS-GBEVs have been closely associated with the virulence of *P. aeruginosa*. Addition of PQS-GBEVs to *pqs* gene mutants of *P. aeruginosa* has been observed to cause a significant increase in the production of virulence factors.^54^ Cooke et al. have revealed that PQS-GBEVs enhanced the dispersion of *P. aeruginosa* biofilms.^43^ The four different stages of *P. aeruginosa* biofilm formation are as follows: attachment stage, wherein *P. aeruginosa* attachment transitions from reversible to irreversible; maturation stage, wherein *P. aeruginosa* growth gradually changes from three-dimensional microcolonies to mushroom-like clusters; dispersion stage, wherein *P. aeruginosa* degrades the outer polymer matrix to expand the living space, and spreads further outward to reach a new attachment surface and begin a new cycle.^70^ Cooke et al. determined that the contents of PQS and GBEVs were significantly higher during the dispersion stage than those during the attachment and maturation stages. During dispersion, GBEVs not only functioned as PQS carriers but also exported various matrix degrading enzymes, including proteases, lipases, and DNAases, to the biofilm to promote the degradation of biofilm matrix components and facilitate *P. aeruginosa* dispersal.^43^ Although PQS-GBEVs may enhance the expansion of *P. aeruginosa* biofilms and further exacerbate the disease in patients infected with *P. aeruginosa*, the use of PQS-GBEVs to precisely regulate biofilm expansion may be an effective therapeutic strategy for converting chronically drug-resistant biofilm infections from recalcitrant protectors to dispersions that are more amenable to antibiotic treatment.

PQS-GBEVs also function in iron transfer. Iron is an essential micronutrient for many living cells and a cofactor for many cellular enzymes.^71^ In the host, iron usually forms high-affinity complexes with proteins to achieve sequestration, thereby enabling the host with resistance to pathogenic infestation since pathogens need iron for metabolism and maintaining virulence.^72^ The main routes of iron uptake by *P. aeruginosa* include the pyoverdine^73^ and pyochelin^74^ iron carriers, both of which bind iron with different affinities and transfer it into *P. aeruginosa* cells *via* TonB receptors, and the Feo system,^75^ which is the most widely distributed ferrous iron transport system in bacteria. Lin et al. constructed a *P. aeruginosa* mutant strain defective in these three iron uptake pathways. Interestingly, they found that this mutant strain could grow in minimal medium containing 0.5 μg/mL iron chelator ethylenediamine-N,N’-bis(2-hydroxyphenylacetic acid).^44^ Subsequently. through genetic screening, the crucial role of *TseF*, a gene close to the type VI secretion system H3 (H3-T6SS), was elucidated. The *TseF* gene encodes a 178-amino acid protein, TseF, which binds directly and specifically to PQS. Moreover, TseF binds to PQS with stronger affinity in the presence of Fe^3+^. TseF is packaged along with PQS into GBEVs, resulting in the formation of TseF-containing GBEVs, which interact with the cell surface Fe (III)-pyochelin receptor FptA and porin OprF, thereby delivering iron molecules into the bacterial cells.^44^

In addition to inducing GBEV secretion by *P. aeruginosa* cells, PQS can also mediate communication across species. Tashiro et al. noticed that the addition of PQS to *E. coli* K12 medium significantly increased the production and size of *E. coli* EVs. For instance, addition of 20 μM PQS increased the size of GBEVs by approximately 20 nm relative to the uninduced group. This effect was also observed in the gram-positive bacterium *Bacillus subtilis* 168, which seldom produces GBEVs under laboratory conditions.^76^

Besides PQS system, three other QS systems for bacteria-bacteria communication have been discovered: the LuxI/LuxR type among gram-negative bacteria,^77^ the oligopeptide-two-component type among gram-positive bacteria,^78^ and the autoinducer 2 (AI-2) type among both gram-negative and gram-positive bacteria.^79^ Each of these QS systems has its own unique regulatory mechanism. In the LuxI/LuxR QS system, acyl-homoserine lactone (AHL) serves as a signal that can only be recognized by related species. In the oligopeptide-two-component QS system, for signaling, autoinducer peptides must be delivered by a dedicated oligopeptide transporter, which is usually an ABC transporter. In the AI-2 QS system, AI-2 is predominantly mediates interspecific communication.^80^ Detection of QS molecules in the host is difficult due to the high volatility and dynamics of QS molecules in the complex intestinal environment. To the best of our knowledge, the phenomena of intestinal bacteria using GBEVs to deliver the three aforementioned QS molecules has not been reported in the literature. Morinaga et al. found that BEVs released by *Paracoccus denitrificans*, a gram-negative, inactive spherical soil bacterium, were able to take up long-chain AHLs secreted by other bacteria from the environment and use it as a signal to control biofilm formation and cell aggregation. This allows *P. denitrificans* to rapidly reach QS threshold concentrations in complex microbial communities with relatively low populations of their own.^81^ In summary, QS molecules can regulate population behavior through their enrichment by BEVs, rather than using the classical diffusion pathway. Therefore, we hypothesize that the use of GBEVs carrying specific QS molecules can regulate bacterial population behavior more precisely. Currently, an important topic in clinical research is the treatment of pathogenic bacterial infections using QS system interfering agents, such as meta-bromo-thiolactone^82^ (inhibits the production of virulence factor pyocyanin and biofilm formation) and cladodionen (inhibits the production of elastase and rhamnolipid, biofilm formation and diffusion, and down-regulates QS-related mRNA expression).^83^ In addition, competitive inhibition of GBEVs carrying pathogenic QS molecules using engineered GBEVs or influencing the formation of GBEVs through interventions (such as the use of bicyclic compounds including 4-hydroxyindole, isatin, 1-naphthol and 8-quinolinol to significantly inhibit the production of *P. aeruginosa* EVs and the synthesis of PQS^84^) are both potential new strategies for the treatment of pathogenic bacteria.

## GBEVs-mediated bacteria-host interactions

**Figure 3.**
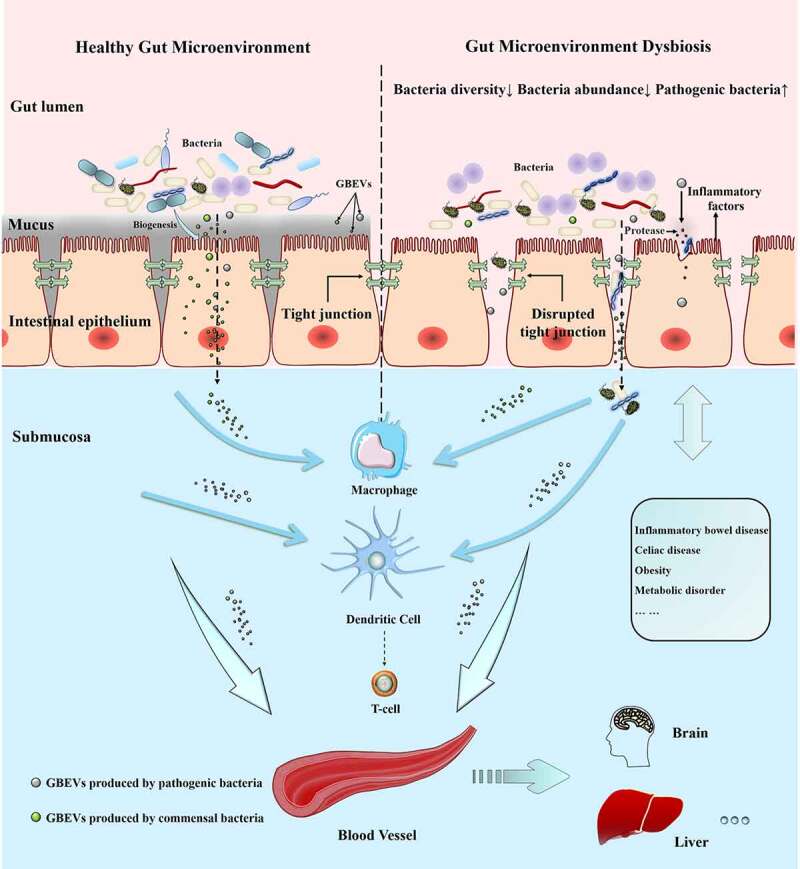
GBEVs mediate cross-talk between gut bacteria and host cells. GBEVs in the gut lumen can access the submucosa. Dysbiosis can lead to the disruption of tight junctions in the intestinal epithelium leading to the liberation of gut bacteria and GBEVs into the underlying submucosa, where GBEVs can activate the immune system and disseminate *via* the circulatory system. GBEVs, gut bacterial extracellular vesicles. GBEVs: gut bacteria-derived extracellular vesicles.

**Figure 4.**
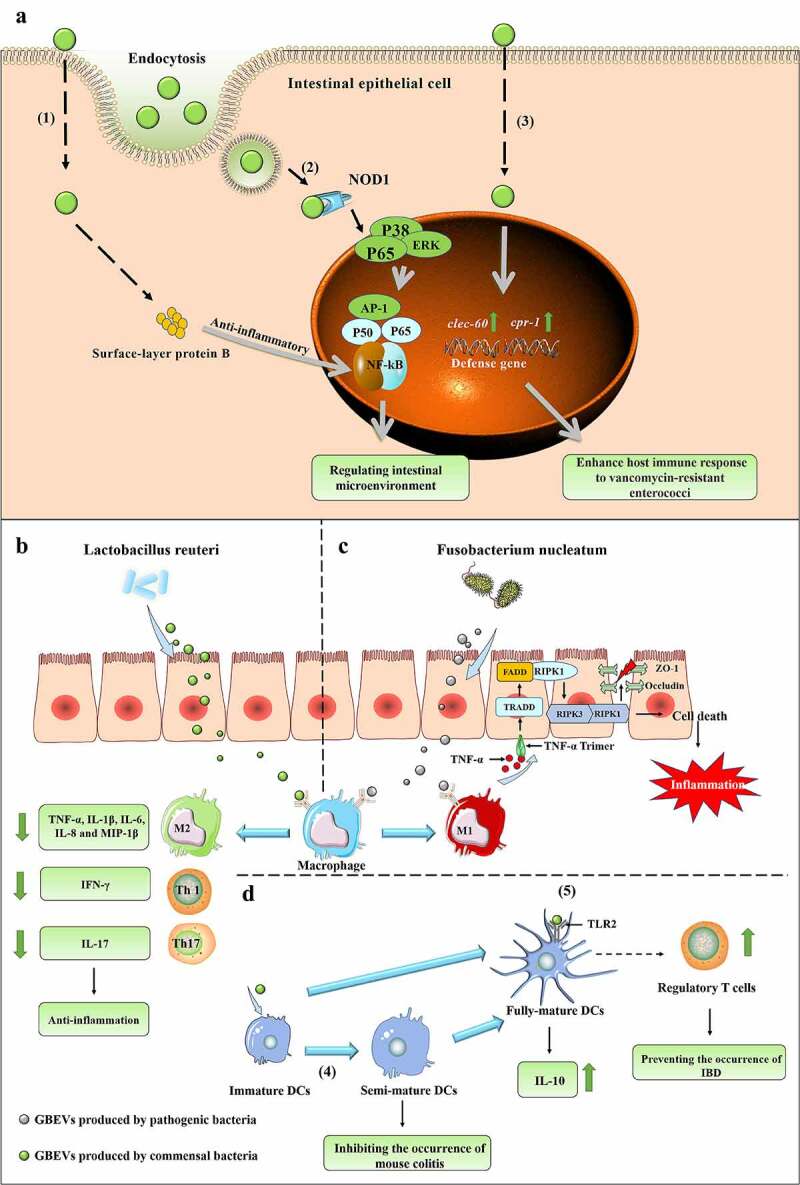
(a) GBEVs mediate communication cross-talk between gut bacteria and intestinal epithelial cells. (1) *Propionibacterium freudenreichii* CIRM-BIA 129 EVs contain surface-layer protein B, which reduces inflammation by regulating the NF-κB pathway in intestinal epithelial cells.^87^ (2) GBEVs (secreted by *Escherichia coli* Nissle 1917) can be internalized by the endocytosis of intestinal epithelial cells and stimulate the intracellular NOD1 receptor, thus regulating the intestinal microenvironment.^88^ (3) GBEVs produced by *Lactobacillus plantarum* WCFS1 up-regulates the expression of host defense genes (*CTSB* and *REG3*) in intestinal epithelial cells and enhances host immune responses to vancomycin-resistant enterococci.^89^ (b) The proposed mechanism of *Lactobacillus reuteri* EV-mediated bacteria-host cross-talk to drive intestinal immune homeostasis against pathogen-induced inflammation in a chicken model.^107^ *L. reuteri* EVs can suppress the pro-inflammatory mediators produced by macrophages. (C) The proposed mechanism of *Fusobacterium nucleatum* EV-driven intestinal mucosal barrier dysfunction in ulcerative colitis.^25^ The release of GBEVs by *F. nucleatum* promotes macrophages to secrete pro-inflammatory factors that activate RIPK1-mediated cell death signals in intestinal epithelial cells, leading to the disruption of intercellular tight junctions.^25^ (D) GBEVs mediate communication cross-talk between gut bacteria and dendritic cells. (4) GBEVs secreted by *Bacteroides vulgatus* can induce DC semi-maturation and enhance the immune system silencing caused by this strain, thus inhibiting the occurrence of murine colitis.^90^ (5) *Bacteroides thetaiotaomicron* and *Bacteroides fragilis* EVs interact with toll-like receptor-2 on dendritic cells (DCs), enhance the production of regulatory T cells and anti-inflammatory cytokines, and induce immune regulation to prevent the occurrence of inflammatory bowel disease.^91,92^

The intestinal microenvironment is primarily maintained by a combination of host commensal microbiota, host cells, and their metabolites. The commensal microbiota provides a variety of benefits to the host, such as obtaining nutrition and energy from food, promoting immune system development, and defense against pathogens.^85^ Previous studies have proved the existence of complex interactions between gut microbiota and the host immune system;^86^ nonetheless, we know that a thick mucus layer between the gut microbiota and intestinal cells limits their direct contact (except in some diseases, such as ulcerative colitis, where damage to the intestinal barrier allows bacteria to cross the mucus layer and make direct contact with IECs). Communication and interaction between gut microbiota and intestinal cells is a rising concern among researchers. GBEVs can directly enter the mucus layer and be endocytosed by host cells (Figure 1B, 3, and 4). Most of the signaling molecules contained within GBEVs are microbe-associated molecular patterns, which can bind to host cell membrane receptors or intracellular receptors, such as LPS (TLR4 ligand), lipoprotein (TLR2 ligand), peptidoglycan (NOD1/NOD2 ligand), DNA, and RNA (TLR7/TLR9 ligand), to regulate host immune responses or promote disease development.^93^ In addition, studies have found that GBEVs of 20–100 nm size enter host cells preferentially through the caveolin-mediated endocytosis pathway, whereas GBEVs of 90–450-nm size enter host cells through the macropinocytosis and endocytosis pathways.^94^ Overall, GBEVs are excellent information carriers that connect bacterial and host cells (Figure 3). Notably, the regulatory effects of GBEVs are strongly dependent on characteristics of the parent bacteria, the cargo loaded, and the host physiology. In this section, we describe the effects of GBEVs on the host (summarized in Table 2). Additionally, some proposed mechanisms underlying GBEV-mediated bacteria-host cross-talk are presented in Figure 4.

**Table 2.** The roles of GBEVs in communication cross-talk between gut bacteria and host cells.

GBEVs source	Cargos	Function	Mechanism	Refs
*Akkermansia muciniphila*	Unknown	1. Protect the progression of dextran sulfate sodium-induced colitis; 2. Increase 5-HT levels in the host intestine.	Unknown	^95–97^
*Bacteroides fragilis*	PSA	Induction of immunomodulation and prevention of experimental colitis.	TLR2	^91^
*Bacteroides thetaiotaomicron*	Unknown	Promote the regulatory DC responses of healthy individuals.	Unknown	^92^
	Sulfatase enzymes	Promote inflammatory immune stimulation in genetically susceptible hosts.	Sulfatase-dependent manner	^98^
	MINPP	Promote intracellular Ca^2+^ signaling.	Unknown	^99^
*Bacteroides vulgatus* mpk	MAMPs	Induce semi-maturation of DCs cells and enhance that immune system silence caused by the strain	TLRs	^90^
*Campylobacter jejuni*	Proteases	Triggering the IL-8, IL-6, hBD-3 and TNF-α responses of T84 intestinal epithelial cells and are cytotoxic to Caco-2 IECs and *Galleria mellonella* larvae.	Unknown	^100^
*Clostridium difficile*	Translation-associated proteins	Inducing a pro-inflammatory response and inducing cytotoxicity of colonic epithelial cells.	Unknown	^101^
*Escherichia coli* BL21	LPS	Activate caspase-11.	TLR4-TRIF-GBPs	^102^
*Escherichia coli* C25	Unknown	It causes mild pro-inflammatory response in intestinal epithelial cells.	TLRs	^93^
*Escherichia coli* Nissle 1917	Unknown	1. Regulation of innate immunity of intestinal epithelial cells; 2. Up-regulation of tight junction protein expression in intestinal epithelial cells.	1. NOD1 2. Unknown	^88,103^
*Escherichia coli* W3110 msbB-mutant strain	Unknown	Induces massive production of IFN-γ and promotes T cell-mediated immune responses.	Unknown	^104^
*Faecalibacterium prausnitzii*	Unknown	Increase 5-HT levels in the host intestine.	Unknown	^96^
*Fusobacterium nucleatum*	1. Autotransporter proteins and other virulence factor (FadA, MORN2 and YadA); 2. Unknown; 3. Unknown	1. Destroy intestinal epithelial barrier; 2. Promote colon cancer development; 3. Regulation of innate immunity of intestinal epithelial cells.	1. FADD-RIPK1-caspase 3; 2. Unknown; 3. TLR2	^25,105–107^
*Lacticaseibacillus paracasei*	Unknown	Inhibit colon cancer development.	PDK1/AKT/Bcl-2	^108^
*Lactobacillus plantarum*	Unknown	Up-regulate the expression of host defense genes to provide protection for the host	Unknown	^89^
*Lactobacillus reuteri*	Glucosyltransferase, serine protease and elongation factor Tu	Enhancing immunomodulatory cell-mediated immunosuppression by activating macrophages to inhibit Th1 and Th17-mediated inflammatory responses	Unknown	^109^
*Pediococcus pentosaceus*	Unknown	Promote that M2-type polarization of macrophages	TLR2	^110^
*Propionibacterium freudenreichii* CIRM-BIA 129	SlpB	Modulate inflammatory responses	NF-κB	^87^
*Pseudomonas aeruginosa*	Unknown	Induces mitochondrial dysfunction in macrophages and inhibits host protein synthesis, ultimately inducing an inflammatory response	Unknown	^111^
*Salmonella* Typhimurium ATCC 14028	Unknown	Inhibit colon cancer development	Unknown	^112^
*Vibrio cholerae* O395	Unknown	Activate innate immune response of host	NOD1	^113^

Notes: DCs, dendritic cells; HFD, high-fat diet; GBPs, guanylate-binding proteins; MAMPs, microbe-associated molecular patterns; MINPP, mammalian InsP6 phosphatase; OMV, outer membrane vesicles; PSA, polysaccharide; SlpB, surface-layer protein B; TLRs, Toll-like receptors; TRIF, TIR domain-containing adaptor-inducing interferon-β; GBEVs: gut bacteria-derived extracellular vesicles; Refs: references.

### GBEVs effects on intestinal barrier function

The intestinal barrier is essential for maintaining the balance of the intestinal microenvironment; it regulates the flow of water, ions, and macromolecules in the lumen.^114^ The intestinal epithelium is firmly bound using tight junction proteins, forming a mechanical barrier to the intestine, which is the main location for digestion and nutrient absorption, allowing the body to achieve a surface area in contact with the external environment of approximately 200 m^2^.^115^ Impaired intestinal barrier function, particularly the intestinal mechanical barrier, can lead to the direct contact of the host cells with intestinal lumen contents, intestinal commensal bacteria, and foreign pathogens, ultimately leading to the onset or development of IBD.^114^ Recent studies have found that the effect of GBEVs on the intestinal barrier varies based on their origin. Most of the GBEVs produced by pathogens impair intestinal barrier function, a property closely related to the parent bacteria. In contrast, GBEVs secreted by intestinal commensal and probiotic bacteria mostly improve intestinal barrier function and suppress the level of intestinal inflammation. Therefore, studying the effects of GBEVs on intestinal barrier function can help design effective interventions aimed at preventing and treating IBD.

#### GBEVs disrupt the intestinal barrier and induce or aggravate IBD

GBEVs can promote the development and progression of IBD by directly inducing apoptosis in IECs. *Fusobacterium nucleatum*, a gram-negative bacterium closely associated with preterm birth, periodontal disease, IBD, and colorectal cancer,^116^ secretes GBEVs that promote macrophage polarization to the M1 type and induce apoptosis in IECs *via* FADD-receptor interacting protein kinase 1 (RIPK1)-caspase 3 pathway, disrupting the intestinal epithelial barrier (Figure 4C).^25^ Liu et al. co-incubated *F. nucleatum* EVs with peripheral blood mononuclear cells and found that it induced a significant increase in the expression levels of pro-inflammatory factors TNF-α and interferon gamma (IFN-γ), inhibited the expression of anti-inflammatory factor IL-10, and polarized macrophages to the M1 type. In the macrophage/colorectal adenocarcinoma (Caco-2) cells co-culture model, treatment of *F. nucleatum* EVs in the presence of macrophages significantly promoted apoptosis of Caco-2 cells and significantly reduced transepithelial electrical resistance (TEER), resulting in the loss of epithelial barrier function.^25^ Moreover, the addition of TNF-α neutralizing antibody or necrostatin-1, a phosphorylated RIPK1 kinase inhibitor, significantly increased Caco-2 cell survival and restored intestinal epithelial barrier function (increased TEER values), suggesting that RIPK1 and RIPK3 in IECs may be involved in the apoptotic process. In addition, these findings were verified using a dextran sodium sulfate (DSS)-induced colitis mouse model. The GBEVs-treated group showed shorter colonic lengths, more severe damage to crypt and epithelial structures, and lower survival rates (100 % survival in the DSS model group and only 50 % survival in the 50 μg/d *F. nucleatum* EVs-treated group) compared with those showed by the DSS model group.^25^ Another study on *F. nucleatum* also demonstrated that the bacteria secretes GBEVs that promote intestinal inflammation. Engevik et al. added *F. nucleatum* subspecies *polymorphum* EVs to HT29 cell (a human colorectal adenocarcinoma cell line) monolayer, which resulted in the significant promotion of IL-8 production.^117^ Interestingly, *F. nucleatum* EVs did not disrupt the intestinal epithelial barrier, although cytokine production and inflammatory responses were observed, which may be strain-dependent. Additionally, clinical isolates from patients with IBD have shown an increased ability to invade Caco-2 cells and induce TNF-α expression than those isolated from healthy populations.^118^ Thus, the virulence of secreted GBEVs varies among different strains of the same species. Remarkably, the virulence mechanism of GBEVs may not be in good agreement with that of parental bacteria. The protein components of *Clostridium difficile* EVs are mainly transcription-related proteins, and no toxic proteins, such as TcdA or TcdB, are contained in the parental bacterial. However, co-incubation of *C. difficile* EVs with Caco-2 cells and HEp-2 cells induces cell death.^101^

In addition to disrupting the intestinal epithelial barrier by inducing apoptosis in IECs, GBEV cargo can affect intestinal barrier function. Wang et al. incubated *E. coli* BL21 EVs with Caco-2, HT-29, and NCM460 cells, and found that GBEVs reduced E-cadherin expression in cells in a concentration-dependent manner.^119^ The team further found that internalization of *E. coli* BL21 EVs by IECs promotes the recruitment of caspase-5 and PIKfyve (involved in endosome maturation) to early endosomal membranes by sorting nexin 10 (SNX10, a member of the SNX family proteins), while promoting the release of LPS from GBEVs into the cytosol. Activated caspase-5 also leads to the phosphorylation of Lyn, the upstream regulator of Snail/Slug, which promotes nuclear translocalization of Snail/Slug and downregulates E-cadherin expression, ultimately leading to intestinal barrier dysfunction. In addition, knocking out *SNX10* using CRISPR/Cas9 or treating with the *SnX10* inhibitor DC-SX029 blocks LPS release, caspase-5 activation, and downstream signaling, effectively alleviating intestinal barrier dysfunction induced by *E. coli* BL21 EVs.^119^ Although the *E. coli* BL21 used in this study is not a typical strain in the host gut, these findings are significant for research on IBD pathogenesis. Apart from LPS, GBEVs secreted by some enteropathogenic bacteria contain protein hydrolases. These GBEVs are internalized by IECs and directly cleave intracellular tight junction proteins, thereby facilitating bacterial-host cell interactions. Elmi et al. analyzed protein fractions in the GBEVs of *Campylobacter jejuni*, the major pathogen of bacterial and foodborne gastroenteritis worldwide, and found three proteases, HtrA, Cj0511, and Cj1365c, responsible for the proteolytic activity of *C. jejuni* EVs (GBEVs produced by *HtrA, Cj0511* and *Cj1365c* mutant strains as well as pretreatment with serine protease inhibitors reduced the protease activity of *C. jejuni* EVs). Co-incubation of *C. jejuni* EVs with T84 monolayers revealed their ability to degrade E-cadherin and occludin in cells in a dose- and time-dependent manner, thereby promoting the adhesion and invasion of *C. jejuni*.^100^ In summary, it is clear that the ability of GBEVs to disrupt the intestinal barrier is inextricably linked to the properties of their parental bacteria. Based on this characteristic, the development of anti-vesiculation drugs will be another new potential strategy for the treatment of IBD.

#### GBEVs modulate host immune system and intestinal barrier to improve IBD

In the intestinal tract, GBEVs secreted by commensal bacteria (including probiotics), may be effective in preventing and treating IBD by modulating the host immune system and intestinal barrier. GBEVs improve IBD by modulating macrophages and dendritic cells (DCs). *Pediococcus pentosaceus* is an intestinal commensal lactic acid bacterium with potent anti-inflammatory activity.^120^ Bulut et al. found that *P. pentosaceus* EVs promote the polarization of marrow-derived macrophages to M2-like macrophages with the involvement of TLR2 receptors, and this anti-inflammatory activity has been validated in both the yeast polysaccharide-induced mouse peritonitis model and the DSS-induced acute colitis mode.^110^ GBEVs secreted by *Bacteroides vulgatus* can induce DC semi-maturation and enhance immune system silencing caused by this strain, thereby inhibiting the occurrence of murine colitis (Figure 4D).^90^ Probiotic *E. coli* Nissle 1917 EVs activate DCs to initiate a Th1 response essential for fighting pathogen infections, and GBEV programmable DCs secreted by the intestinal commensal bacteria ECOR12 coordinate the production of a T-regulatory response essential for sustained immune tolerance in the intestine.^121^

Apart from directly interacting with immune cells, GBEVs can also alleviate intestinal inflammation by augmenting the endoplasmic reticulum stress response. *Lactobacillus paracasei* is an important Lactobacilli, and clinical studies have found a link between a reduction in *Lactobacillus* spp. in the gut and the increased incidence of IBD.^122,123^ Choi et al. applied *L. paracasei*-derived GBEVs to LPS-stimulated HT29 cells and DSS-induced colitis mice model. The results showed that *L. paracasei* EVs significantly reduce the levels of LPS-induced inflammatory factors (IL-1α, IL-1β, IL-2, and TNF-α), inflammation-related proteins (cyclooxygenase-2, inducible nitric oxide synthase, and nuclear factor kappa B) and nitric oxide (molecules strongly associated with IBD severity), and significantly improve the symptoms of DSS-induced colitis. In contrast, the addition of endoplasmic reticulum stress inhibitors, salubrinal or 4-phenylbutyric acid, or CHOP siRNA block the anti-inflammatory effect of *L. paracasei* EVs in LPS-stimulated HT29 cells and restore the LPS-induced inflammatory response.^124^ In addition to exerting immunomodulatory effects, GBEVs can also ameliorate IBD by modulating the structural proteins of IECs. For example, GBEVs secreted by *E. coli* Nissle 1917 and intestinal commensal strain ECOR63 promote the upregulation of tight junction proteins ZO-1 and claudin-14 in IECs, downregulate claudin-2 levels, enhance the intestinal barrier, and reduce intestinal permeability.^103^

While some GBEVs can improve IBD by directly stimulating the host immune system or modulating the intestinal mechanical barrier, the ameliorative effect of GBEVs on IBD is majorly achieved by the cargos they carry. Hu et al. found that *Lactobacillus reuteri* BBC3 EVs carry nucleic acid molecules as well as a variety of bioactive proteins, such as glucosyltransferase (glycosylation of host target proteins, activation of DC-SIGN signaling, induction of IL-10 production),^125^ serine protease (selective targeting of pro-inflammatory factors to suppress the inflammatory response)^126^ and elongation factor Tu (involved in intercellular signaling).^99^ The administration of GBEVs to LPS-stimulated broilers significantly lessened the reduction in body weight and increased food intake and effectively reduced mortality. Measurement of pro- and anti-inflammatory gene expression levels in jejunum tissues revealed that *L. reuteri* BBC3 EVs significantly inhibit the expression of pro-inflammatory genes (*TNF-α, IL-1β, IL-6, IL-8*, and *IL-17*) and promote the expression of anti-inflammatory genes (*IL-10* and *TGF-β*), thereby reducing LPS-induced intestinal inflammation. *In vitro* cellular experiments revealed similar results, wherein GBEVs were internalized within 6 h of co-incubation with chicken HD11 macrophages. GBEVs pretreatment significantly inhibited NF-kB expression, reduced *TNF-α, IL-1β*, and *IL-6* gene expression, and enhanced *IL-10* and *TGF-β* gene expression in LPS-stimulated HD11 cells (Figure 4B). However, the addition of DNase I (10 U/mL), RNase I (10 U/mL), and proteinase K-agarose (1 mg/mL) significantly attenuated the inhibitory effect of *L. reuteri* BBC3 EVs on the LPS-induced inflammatory response.^109^ Although no specific effector substances were identified in this study, identifying substances within GBEVs that are involved in the regulation of host immune responses was possible. Polysaccharide is an immunomodulatory factor produced by *B. fragilis*, and Shen et al. found that *B. fragilis* can deliver polysaccharide to immune cells *via* GBEVs, thereby preventing IBD. *B. fragilis* EVs are endocytosed by DCs after entering the body and interact with polysaccharide *via* TLR2 receptors to enhance the production of regulatory T cells and anti-inflammatory cytokines and induce immune regulation (Figure 4D).^91^ *Propionibacterium freudenreichii* CIRM-BIA 129 EVs contain a large amount of surface-layer protein B (SlpB), which interacts with the immunomodulatory transcription factor NF-κB through protein-protein interactions. Co-incubation of GBEVs with HT-29 cells revealed that *P. freudenreichii* CIRM-BIA 129 EVs inhibit LPS-induced IL-8 secretion, and this anti-inflammatory effect is dependent on SlpB, which is not eliminated by the proteinase K-induced hydrolysis of EV surface proteins, suggesting that SlpB is present within GBEVs (Figure 4A).^87^

Overall, the effects of GBEVs secreted by intestinal pathogenic bacteria, intestinal commensal bacteria, and probiotic bacteria on IBD are all based on their corresponding parental bacteria. However, it is worth noting that we cannot equate the effect of GBEVs on IBD with the effect of parental bacteria on IBD. Kang et al. revealed that the pretreatment of CT26 cells with *Akkermansia muciniphila* EVs significantly reduced IL-6 levels produced under *E. coli* EV induction. The oral administration of *A. muciniphila* EVs was also shown to improve DSS-induced phenotypic changes in IBD in animals. However, the direct treatment of DSS mice with *A. muciniphila* did not have a therapeutic effect, instead it exacerbated the colitis.^95^ This suggests that GBEVs may affect the host differently than the parental bacteria, and these effects are not uniquely beneficial or detrimental and need to be judged in the context of the intestinal environment. Collectively, these findings suggest that GBEVs may be a double-edged sword, the consequences of which need to be further explored.

### GBEVs regulate colon cancer occurrence and progression

Globally, colon cancer is one of the most common tumors. Statistically, it ranks third and second in incidence and mortality, respectively, among all cancer types.^127^ Imbalance in the intestinal microenvironment is an important factor in the development and progression of colon cancer.^128^ According to 16S rRNA sequencing, compared with healthy individuals, patients with colon cancer manifest significantly lower gut flora diversity and abundance.^129^ In addition, researchers have found that *F. nucleatum*, genotoxic *E. coli*, and enterotoxigenic *B. fragilis*, all promote the development of colon cancer in the animals.^128^ GBEVs, as important bacteria-bacteria and bacteria-host communication agents, also play an important role in the occurrence and development of colon cancer. Recently, Park et al. performed a metagenomic analysis of GBEVs in the stool samples of 70 patients with colon cancer and 158 control participants and found significant differences in the composition and diversity of GBEVs. The GBEV composition in the stool of patients with advanced colon cancer was significantly altered relative to that of patients with early colon cancer. Among these, GBEVs secreted by bacteria belonging to the genus *Alistipes*, a member of *Bacteroidetes* phylum, first reported in 2003, and linked to liver fibrosis, IBD, cancer, and cardiovascular disease^130^ may be a biomarker for colon cancer staging.^131,132###^

In addition to the association of GBEVs with colon cancer progression, studies have suggested that *F. nucleatum*-secreted GBEVs are also involved in colon cancer development. Tai et al. co-cultured *F. nucleatum* EVs with Caco-2 cells and found that it promotes mitochondrial fusion and invasiveness in cells, while the use of *Paris polyphylla*, an herb, inhibits the spread of colon cancer.^105^ Using enzyme-linked immunosorbent assay (ELISA), Lamprinaki et al. determined that LPS-loaded *F. nucleatum* ssp. animalis EVs could bind to sialic acid-binding immunoglobulin-like lectins-7, which are abundant on innate immune cells and tumor infiltrating T cells and inhibit immune activation upon binding to LPS, and GBEVs induced TNF-α production by human monocyte-DCs, thereby creating a pro-inflammatory environment to promote colon cancer development.^133^

Some bacteria secrete GBEVs that promote colon cancer progression, whereas some naturally occurring GBEVs induce apoptosis in colon cancer cells to exert antitumor effects. Shi et al. found that *Lacticaseibacillus paracasei* EVs have an inhibitory effect on colon cancer growth. GBEVs are absorbed by colon cancer cells (HCT116, SW1116, and SW620 cells) and significantly inhibit colon cancer cell proliferation, migration, and invasion in a concentration-dependent manner.^108^ *L. paracasei* EVs (200 μg/mL), upon co-incubation with colon cancer cells for 48 h, significantly promote their apoptosis, as determined using Annexin V/PI double staining. The xenograft tumor (seeding HCT116 cells) mice assay has shown extremely slow tumor growth in the GBEVs-treated group. Studies on its antitumor mechanism revealed that *L. paracasei* EVs significantly inhibit 3-phosphoinositide-dependent protein kinase-1 (serine tryptophan kinase, involved in signal transduction) and *AKT* (serine-threonine kinase, involved in the regulation of cell survival, proliferation, and apoptosis) genes and decreased the expression of Bcl-2 protein (an anti-apoptotic protein) in colon cancer cells.^108^ Similar effects have been observed with *S*. Typhimurium ATCC 14028 EVs.^112^ Although these studies demonstrated the anti-colon cancer effect of some GBEVs, none of them identified which specific molecules were responsible for the anti-tumor effect. Since the cargos of GBEVs secreted by bacteria vary with time period and environment, strategizing to effectively achieve the quality control of GBEVs with anti-colon cancer effect is critical.

Apart from directly inducing apoptosis in colon cancer cells, GBEVs can also be used as an advanced immune irritant to effectively induce *in vivo* anti-tumor immune responses for cancer treatment. Kim et al. injected *E. coli* BL21 and *E. coli* W3110 *msbB*-mutant-derived EVs (*msbB*, the gene encoding lipid A acyltransferase) into the tail vein of colon adenocarcinoma CT26 mice and found that GBEVs could be specifically targeted and enriched in tumor tissue, with extended anti-tumor activity by inducing massive IFN-γ production and promoting T-cell-mediated immune responses. In addition, GBEVs also exhibit therapeutic effects on other tumors; intravenous injection into mice inoculated with 4T1 murine carcinoma and B16BL6 melanoma cells significantly inhibits tumor growth and metastasis.^104^

Currently, colon cancer research is inclined towards considering its role in terms of EVs of host cell origin, neglecting the large number of GBEVs of intestinal bacterial origin. Moreover, our knowledge of how GBEVs affect colon cancer is extremely limited. For example, it is known that *B. fragilis*^134^ and its secreted toxin (BFT)^135^ can promote colon cancer development; however, regarding *B. fragilis* EVs, it is only known that they can improve IBD. Whether BFT can be loaded and whether it can affect the occurrence and progression of colon cancer are remain unclear. Recently, some studies have also found that probiotics function as disruptors in cancer development. For instance, *Lactobacillus* spp. promote the growth of pancreatic cancer tumors in mice.^136^ However, the role of GBEVs, which act as bacteria-host communicators, therein needs to be further investigated.

### Immune modulation by GBEVs

Intestinal bacteria can contribute to the maturation and development of the host immune system through GBEVs. The IEC barrier is an important host defense mechanism against infection by exogenous pathogens and is the first site of interaction between GBEVs and the host. Once GBEVs cross the epithelial barrier into the submucosa, they interact with various immune cells, thereby regulating the host’s immune system. Kaparakis-Liaskos and Ferrero have provided a complete summary of how bacterial GBEVs modulate the host immune system.^137^ Hence, this section focusses on the recently reported modulation of the host immune system and intestinal microenvironment by intestinal pathogenic bacteria-, intestinal commensal bacteria-, and probiotics-secreted GBEVs.

#### GBEVs interactions with IECs

The regulatory role of intestinal bacteria on IECs innate immune system (rapid response to intestinal pathogenic microorganisms and slow response to intestinal commensal and probiotic bacteria) is an important factor in maintaining homeostasis of the intestinal microenvironment.^138^ GBEVs also play an important role in this regard by activating the innate immunity of IECs through various pathways, thus promoting the host defence against pathogenic bacteria and maintaining stability of the intestinal microenvironment.

GBEVs modulate the innate immunity of IECs by interacting with TLR2 receptors. Martin-Gallausiaux et al. exposed Caco-2 cells in a non-inflammatory environment to *F. nucleatum* EVs (10 μg/mL). Epithelial resistance of the cells was measured and found that *F. nucleatum* EVs did not disrupt the barrier of IECs and had no effect on the expression levels of the tight junction proteins occludin and claudin-2. In contrast, after the co-culture of *F. nucleatum* EVs with T84 cells, GBEVs induced activation of the NF-kB pathway and induced production of the inflammatory factor IL-8 (neither Caco-2 nor HT-29 cells showed NF-kB activation).^106^ FomA, a porin which is immunogenic and is involved in bacterial adhesion,^139,140^ is a key substance central to this phenomenon. The investigators used anti-FomA binding peptide to pretreat GBEVs, which effectively reduced their activity, while NF-kB expression was also significantly reduced after TLR2 was blocked in T84 cells using siRNA. This suggests that both FomA and TLR2 are involved in the activation of NF-kB pathway by *F. nucleatum* EVs.^106^ In previous studies, *F. nucleatum* EVs were generally found to have adverse effects on the host, such as exacerbating IBD or promoting colon cancer development. However, when the IECs were placed under steady-state conditions, *F. nucleatum* EVs did not cause pathological inflammatory responses and activated the host innate immunity by interacting with TLR2. Thus, the effects of GBEVs on the host cannot be categorized as “good” or “bad”, but are inextricably linked to the host’s physiological state.

In addition to recognizing extracellular TLR receptors, GBEVs can enhance innate immunity by activating intracellular nucleotide oligomerization domain 1 (NOD1) receptors in IECs through the peptidoglycan they carry. The NOD1 receptor is a member of the NOD-like receptor family of innate immune proteins, which is expressed in a variety of cell types and is particularly highly expressed in IECs.^141^ The NOD receptor consists mainly of the N-terminal caspase-activated recruitment domain (involved in protein interactions), the central NACHT domain (involved in receptor dimerization), and the C-terminal leucine rich repeat domain (which recognizes specific ligands).^142^ It can be activated by bacterial peptidoglycan fragments, thereby acting as an intracellular sensor of bacterial infection. Canas et al. incubated *E. coli* Nissle 1917 and GBEVs secreted by the intestinal commensal bacterium ECOR12 with IECs and determined, through confocal fluorescence microscopy, that GBEVs could be internalized by IECs through endocytosis. Internalized GBEVs further trigger the formation of NOD1 aggregates in IECs, which activates the NF-κB signaling pathway and promotes the expression of inflammatory factors IL-6 and IL-8 (Figure 4A).^88^ While the sustained stimulation of NOD1 by GBEVs from both sources results in an inflammatory response, GBEVs secreted by *E. coli* Nissle 1917 and Commensal ECOR63 promote the expression of tight junction proteins in IECs, thereby improving IBD.^103^ Therefore, the biological effects of GBEVs should be evaluated from multiple perspectives, and should not be judged by a single indicator. Thus, NOD1 receptors in IECs can activate GBEVs produced by probiotic and intestinal commensal bacteria; GBEVs secreted by *V. cholerae* O395 can also mediate the host innate immune response *via* NOD1 receptors. Chatterjee and Chaudhuri co-cultured 50 μg/mL *V. cholerae* O395 EVs with Int407 cells (the most common cell model for studying *V. cholerae*-host interactions) and HEK293 cells and found that the levels of pro-inflammatory IL-8 and granulocyte-macrophage colony-stimulating factor (GM-CSF) were significantly elevated.^113^ The investigators also observed that NOD1 expression was significantly upregulated in HEK293 cytoplasm, while NOD1, IL-8, and GM-CSF expression levels were significantly reduced after treatment with NOD1-specific siRNA. In the co-culture system of HT-29 and DCs, the expression of co-stimulatory molecules (HLA-DR, CD80, and CD83) on the surface of DCs was significantly upregulated by adding *V. cholerae* O395 EVs to stimulate HT-29 cells, indicating that *V. cholerae* O395 EVs can activate DCs. Based on this, the team added naive CD4^+^CD45RA^+^ T cells purified from peripheral blood to the above culture system for 5 d. The results of ELISA indicated that IL-4, IL-13, and IL-17 levels were significantly upregulated and IFN-γ expression levels were significantly downregulated, indicating that the T cell population was stimulated by *V. cholerae* O395 EVs to Th2/Th17 and the responses were polarized.^113^ Overall, this study demonstrated, at the *in vitro* cellular level, that *V. cholerae* O395 EVs induce inflammatory responses and activate host innate immune responses *via* the NOD1 pathway. Notably, the regulation of host innate immune responses by GBEVs secreted by different serotypes of *V. cholerae* may also differ; for example, the secreted GBEVs of *V. cholerae* V:5/04 non-O1 non-O139 clinical isolate,^47^ fail to activate host innate immune responses and promote strain colonization by suppressive effects.

GBEVs can protect the host from pathogens by activating the innate immune system of IECs and upregulating the expression of host defense genes in IECs. Li et al. evaluated the protective effect of *Lactobacillus plantarum* WCFS1 EVs using *Caenorhabditis elegans* and Caco-2 cells attacked by *Enterococcus faecium* (VRE) as a model. The solid killing assay revealed that nematodes pretreated with *L. plantarum* WCFS1 EVs survived the VRE attack for approximately 4 d longer than that by the control, and their defense genes *cpr-1* (expressing gut-specific cysteine protease) and *clec-60* (expressing C type lectin) were upregulated approximately 3-fold and 9-fold, respectively. Similarly, *L. plantarum* WCFS1 EVs co-incubated with Caco-2 cells for 24 h did not show cytotoxicity and result in a significant upregulation of *CTSB* (cathepsin B) and *REG3* (regenerating islet-derived protein 3-gamma), which are functionally similar to *cpr-1* and *clec-60* genes (Figure 4A).^89^

#### Effects of GBEVs on neutrophils

Neutrophils are an important part of the host’s innate immune system. During bacterial infection, neutrophils are rapidly recruited to the infection site, and they swiftly clear the infection by secreting hydrolytic enzymes, proteinases, reactive oxygen species (ROS), chemotactic agents, and other antimicrobial substances.^143^ GBEVs are involved in regulating memory-like inflammatory responses of neutrophils, with dose-dependent variations in immune responses. Hudalla et al. co-incubated fecal-derived GBEVs with mouse bone marrow-derived neutrophils and found that GBEVs directly mediate adaptive immune responses.^144^ Pro-inflammatory sensitivity of neutrophils induced by low doses of GBEVs (1 ng/mL) is significantly enhanced, and TNF-α, IL-6, and ROS levels are significantly upregulated after the second hit of LPS. In contrast, increasing the dose of GBEVs to 28.1 μg/mL leads to an immune tolerance phenotype (diminished response to LPS restimulation) in neutrophils and promotes the expression of the anti-inflammatory factor IL-10.^144^ Of note, since the source of GBEVs in this study was feces, it may be difficult to separate host cell EVs and GBEVs in fecal samples by centrifugation and qEV columns alone.

#### Modulation of macrophages and DCs by GBEVs

Except for the above mentioned GBEVs that can improve IBD by modulating macrophages and DCs, not many studies have been conducted on GBEVs interacting with macrophages and DCs to affect the host immune system. In terms of macrophages, Deo et al. found that *P. aeruginosa* EVs activate macrophages-mediated inflammatory responses. The team co-cultured *P. aeruginosa* EVs with macrophages and found that they induced mitochondrial dysfunction and inhibited host protein synthesis, ultimately activating intrinsic apoptosis and inflammatory responses.^111^ In a study of the interaction between GBEVs and DCs, Durant et al. found that *B. thetaiotaomicron* EVs are involved in regulating DCs-mediated immune responses. They co-incubated DCs from the colonic mucosa and blood of healthy volunteers with *B. thetaiotaomicron* EVs and measured the cytokines secreted by the DCs. Interestingly, *B. thetaiotaomicron* EVs produced different effects on DCs from different sources, stimulating significant expressions of regulatory IL-10 and protective IL-6 for DCs of colonic and peripheral blood origin, respectively (Figure 4D). Thus, *B. thetaiotaomicron* EVs balance DCs-mediated immune responses when the host is in a healthy state.^92^ The investigators also used *B. thetaiotaomicron* EVs to stimulate DCs in patients with IBD (Crohn’s disease and ulcerative colitis); however, the above effects completely disappeared. This suggests that the different physiological states of the host may also affect the response of DCs to GBEVs.

### GBEVs regulate serotonin levels and affect intestinal microenvironment stability

Serotonin (5-HT) is an important signaling molecule in the body, which transmits signals from the intestinal lumen to the lateral neurons, thus participating in synaptic signaling in the nervous system. In addition, 5-HT is a key molecule in maintaining the stability of the intestinal environment by regulating the secretory and motor functions of the gut.^145^ Approximately 95 % of 5-HT in humans is localized in the gastrointestinal tract and is mainly synthesized by enterochromaffin cells in the intestinal mucosa with the help of the enzyme tryptophan hydroxylase 1 (TpH1).^146^

Recently, Yaghoubfar et al. found that GBEVs can alter 5-HT levels in the host intestine.^96^ By co-incubating bacteria with GBEVs, Yaghoubfar et al. revealed that the pretreatment of CT26 cells with *A. muciniphila* EVs and *Faecalibacterium prausnitzii* EVs significantly increased 5-HT levels, expression of *Slc6a4* (involved in serotonin transport) and *Tph1* (rate-limiting enzyme associated with serotonin biosynthesis), while decreasing the expression of *Mao* gene (associated with serotonin metabolism). Curiously, the parental strains of both only affected the expression of *Slc6a4* (significantly increased) and *Mao* (significantly decreased) genes, with no effect on serotonin levels in cells and *Tph1* expression.^96^ Another study by the team on *A. muciniphila* and its GBEVs found that 5-HT levels in both the hippocampus and colon of C57BL/6 J mice significantly increase after 5 weeks of administering *A. muciniphila* and its EVs. The mRNA levels of the *Tph1* gene in the colon are also significantly increased after treatment with both *A. muciniphila* and its GBEVs.^97^ Although the effects of *A. muciniphila* differed in the two studies, the effects of its secreted GBEVs were similar in both cases. Unfortunately, neither of these studies further investigated the biological effects in the host after the rise in 5-HT caused by GBEVs. As 5-HT is both a hormone and a neurotransmitter, it has multiple effects on both the intestinal microenvironment and the central nervous system, and the role of GBEVs in the regulation of 5-HT needs to be further explored.

In addition to regulating host intestinal microenvironment *via* the above pathways, GBEVs can also affect lesions involving distal host organs *via* the gut-brain axis, gut-liver axis, and other pathways (Figure 3). This part of the content is not the focus of this review. We recommend that readers interested in the topic of “GBEV-mediated gut-distal organ communication” to refer to an in-depth review of the gut-brain axis^147^ and gut-liver axis.^148^ In the following section, we describe the potential medical applications of GBEVs.

## Repurposing GBEVs in medical applications

### GBEVs as vaccines

The activation of host innate and adaptive immune responses by GBEVs secreted by pathogenic intestinal bacteria allows the development of GBEVs as a low-toxicity, highly effective vaccine or adjuvant. *S. typhimurium* is the main acute gastroenteritis-causing pathogen. Schetters et al. found that *S. typhimurium* EVs can induce the maturation of human monocyte-derived DCs, mouse bone marrow-derived DCs, and CD11c^+^ splenic DCs, which in turn activate CD8^+^T cell responses. The CD8^+^T cell response is currently a key clinical treatment strategy for tumors and intracellular viruses. Therefore, *S. typhimurium* EVs could be developed as an effective vaccine to inhibit viral replication and tumor growth.^149^ Enteric bacterial infections are a leading cause of diarrhea. Statistically, in developing countries, up to 10% of deaths in children under five years of age are caused by diarrhea.^150^ *Shigella boydii* is a major diarrhea-causing pathogen in children, and approximately 165 million cases have been reported annually worldwide. Although antibiotics are an effective strategy to control the outbreak of shigellosis, they are accompanied by the continuous emergence of antibiotic-resistant *Shigella*. Mitra et al. found that *S. boydii* EVs can significantly inhibit *S. boydii*-mediated inflammation and enable prolonged survival of infected mice.^151^

Introducing and expressing heterologous genes into non-pathogenic engineered *E. coli* is also a novel strategy for preparing a low-toxicity, well-controlled, and high-yield GBEV-based vaccine. In 2004, Kesty et al. first demonstrated that heterologously expressed outer membrane and periplasmic proteins can be incorporated into GBEVs.^152^ These findings enable the production of “customized” GBEVs. Price et al. introduced a plasmid containing a gene encoding the structure of glycan (derived from *C. jejuni*) into *E. coli* for expression, and the secreted GBEVs contained a large amount of glycan molecules identical to the pathogenic bacterial surface glycan (*C. jejuni* heptasaccharide N-glycan).^153^ The test in a *C. jejuni* attack model revealed that the numbers for colonization by *C. jejuni* in chicken intestines inoculated with GBEVs was nearly 104-fold lower than that in a model group.^153^ Guido et al. introduced a heterologous antigen gene with a fused lipoprotein leader sequence into *E. coli*, successfully synthesized the heterologous antigens by lipid modifications, and realized high-level accumulation in GBEVs (accounting for 5–20% of the total protein content of GBEVs) at the same time. In addition, the expression of lipidated xenoantigens also interfered with the acylation of lipid A, so as to significantly reduce LPS-mediated reactivity. This preparation strategy was verified using five *Staphylococcus aureus* antigens, and mice immunized with the engineered GBEVs could ultimately tolerate pathogenic attacks by *S. aureus*.^154^

### GBEVs as delivery vehicles

Owing to their small size and natural lipid bilayer, GBEVs can accommodate a variety of biomolecules with different properties, such as lipids, nucleic acids, and proteins, making them an ideal drug delivery system.

#### Modified GBEVs play a therapeutic role

Surface modification of GBEVs improves their targeting ability and reduces their toxic effects. Peng et al. introduced a plasmid containing recombinant human tumor necrosis factor-related apoptosis-inducing ligand (TRAIL) gene into engineered bacteria (*E. coli*) to construct GBEVs containing the active TRAIL protein.^155^ To reduce LPS toxicity in GBEVs, the investigators co-incubated the GBEVs with 2 mg/mL lysozyme for 1.5 h, allowing lysozyme to bind to LPS efficiently. Meanwhile, to achieve tumor targeting, the investigators modified the αvβ3 integrin targeting ligand (RGP) on GBEVs. After applying the modified GBEVs to melanoma sites in mice, the GBEVs fused with the skin through the nano-size effect, thereby efficiently penetrating the dermal stratum corneum of the mice. Upon entry into the skin, GBEVs specifically recognize melanoma cells with the help of RGP, thereby releasing the TRAIL protein and promoting the apoptosis of melanoma cells.^155^ In addition, in order to circumvent antibody-dependent clearance of GBEVs and the high toxicity caused by intravenous injection of GBEVs secreted by *E. coli* BL21, Qing et al. covered the surface of GBEVs with a pH-sensitive calcium phosphate shell, which could neutralize the acidic microenvironment of tumors and cause macrophages to become highly and effectively polarized from M2 to M1 types, thereby improving the antitumor effects of the GBEVs.^156^

#### GBEVs play a therapeutic role as a drug carrier

Exploiting the similarity between GBEVs secreted by pathogenic bacteria and parental bacteria as well as the natural structure of the lipid bilayer, researchers wrapped antibiotics with GBEVs, effectively realizing antibiotic camouflage and intracellular delivery, successfully solving the clinically-difficult problem of intracellular bacterial infection. For example, Gao et al. used *S. aureus* EVs as carriers and wrapped poly (lactic-co-glycolic acid) nanoparticles containing antibiotics by membrane-coating technique (NP@EV).^157^ The efficiency of NP@EV uptake by murine Ana-1 macrophage was examined using a fluorescence microscope and fluorimeter. Subsequently, Gao et al. discovered that Dil-labeled NP@EV was efficiently taken up by macrophages, and relatively more NP@EV was taken up by macrophages infected with *S. aureus*. The investigators also used PEGylated lipid bilayer-coated nanoparticles and *E. coli* EV-coated nanoparticles as controls and found that both were internalized less efficiently by macrophages than that observed for the NP@EV group. To evaluate the *in vivo* bacteriostatic effect of NP@EV, Gao et al. injected NP@EV into *S. aureus* bacteremia-bearing mice *via* the tail vein. NP@EV was significantly enriched in the kidney, lung, spleen, and heart of the mice model compared with that in the normal controls. Moreover, NP@EV reduced bacterial burden in the kidney and lungs by approximately 1–2 orders of magnitude compared with the untreated group, indicating effective alleviation of *S. aureus* metastatic infection.^157^

Some GBEVs can stimulate the host immune system. These GBEVs can be combined with anticancer drugs or formulations by virtue of their characteristics, thereby exerting synergistic therapeutic effects. Chen et al. used polyethylene glycol and tumor-targeting ligand Arg-Gly-Asp peptide to surface-modify GBEVs isolated from attenuated *Salmonella* to improve the circulatory stability and tumor-targeting of GBEVs.^158^ Additionally, micelles loaded with tegafur, a prodrug of fluorouracil that triggers direct apoptosis of tumor cells and also sensitizes tumor cells to cytotoxic T lymphocytes, were encapsulated within GBEVs (ORFT) to improve its inhibitory effect on tumor cells. Studies on *in vitro* cellular uptake of ORFT revealed that both murine macrophages RAW264.7 and B16F10 cells achieved rapid and high levels of ORFT uptake. Studies on *in vivo* efficacy of ORFT have revealed that ORFT injection effectively delays tumor development (tumors appeared at day 13 in the saline group, day 17 in the tegafur-loaded micelles group, and day 21–24 in the ORFT group), prolongs the survival of melanoma mice (from 31 to 39 d), and effectively inhibits tumor metastasis to the lung (significant reduction of pulmonary metastatic melanoma).^158^ Li et al. used *E. coli* Trans T1 EVs to mask cisplatin nanoparticles, exploiting its pathogen-associated molecular pattern, which is similar to that of the parental bacteria, resulting in effective recognition and internalization of these EVs by neutrophils, which were subsequently transferred to the site of tumor inflammation.^159^ Under the stimulation of inflammation, neutrophils rapidly released cisplatin nanoparticles, which were then absorbed by tumor cells and exerted anti-cancer effects.^159^

### GBEVs as diagnostic biomarkers

Diseases are strongly correlated with biological imbalances involving intestinal microbiota. Diagnosing the existence and development of diseases by detecting changes in gut flora is a crucial area of clinical research. Current studies surrounding biomarkers are dominated by detection of changes in microbiota and metabolites in fecal samples; however, in fecal samples, in addition to live bacteria, a significant number of dead bacteria are also present. The possible reasons for bacterial death are numerous and may involve drug-induced inactivation or prolonged exposure of bacteria to air during sampling. The existence of these partially-inactivated bacteria will undoubtedly interfere with the search for disease-related biomarkers, making it difficult to translate research findings into clinical settings.

GBEVs, as information delivery vectors secreted during the lifespan of living bacteria, contain nucleic acid, protein, and/or lipid molecules derived from parental bacteria. By detecting these molecules contained therein to characterize changes in the intestinal microbiota and metabolites of patients, researchers can ascertain biomarkers more accurately. For example, Kim et al. detected a significant increase in the abundance of *Firmicutes* phyla, such as *Eubacterium, Faecalibacterium*, those of the *Ruminococcaceae* family, and *Catenibacterium*, by examining intestinal bacteria and GBEVs isolated from the fecal samples of patients with colon cancer.^160^ Furthermore, Kim et al., showed that GBEVs isolated from patients with severe alcoholic hepatitis have lower α diversity and higher β diversity than those in healthy controls and that the number of GBEVs secreted by *Veillonella sp., V. parvula, and Lactobacillales sp*. were significantly increased.^161^ At present, research regarding GBEV-disease-related biomarkers is at a naive stage. Effective isolation and detection of the number and types of GBEVs in biological samples are of the essence, requiring prompt and precise scrutiny.

## Conclusions and future perspectives

Overall, our review summarized the role of GBEVs in bacteria-bacteria interactions and bacteria-host communication in the gut, indicating the role of GBEVs in intestinal microenvironment regulation. The research on BEVs has made tremendous progress recently. BEVs are now widely recognized as carriers of information which act as communicative agents between bacteria and hosts. However, to date, most studies on BEVs have focused on BEVs secreted by pathogenic bacteria.

Increase in the knowledge pertaining to GBEVs, a number of issues still need to be further addressed. Currently, methods for separation of GBEVs and host exosomes are generally based on physical factors, such as size and density and clearly differentiating between the two using these methods is difficult. Therefore, the results obtained from studies that have employed such methods need to be further explored. For example, Wei et al. found that GBEVs could increase blood-brain barrier permeability, promote the activation of brain astrocytes and microglia, and induce inflammatory responses and tau hyperphosphorylation by activating the GSK‐3β pathway, resulting in cognitive impairment.^162^ However, in this experiment, researchers isolated GBEVs directly from feces without considering the removal of exosomes secreted by mammalian cells. Thus, whether the series of effects observed by Wei et al. can completely be attributed to GBEVs remains unclear. Since the outer surface of GBEVs often contains LPS or lipoteichoic acid, which are specific to bacteria, we speculate that efficient separation of GBEVs and host cell exosomes is possible based on these unique properties.

Cargos are the material basis for the pathogenic or regulatory effects of GBEVs on the host, and clarifying the composition of cargos can provide fundamental information and implementation strategies for the development of GBEVs as potential vaccines and microenvironmental regulators. However, putting this theory into practice still remains challenging due to several issues. The cargo that is loaded and carried within GBEVs is closely related to the growth environment and exogenous stimuli of the parental bacteria. For example, Zavan et al. found that *Helicobacter pylori* secreted different cargos within GBEVs at different growth stages, resulting in different biological effects.^163^ In 2017, the Tashiro group demonstrated, for the first time that, GBEVs can selectively interact with parental bacterial cells in the microbial community. By labeling GBEVs with fluorescent membrane dyes, they found that GBEVs secreted by *Buttiauxella agrestis* specifically interacted more with other *B. agrestis* cells than bacteria of other genera.^164^ Whether such selective intercellular communication exists in other bacteria remains unclear. Another key issue affecting the application of GBEVs is their targeting behavior once they enter a cell (intestinal bacteria or host cell). Therefore, in view of these uncertainties about naturally occurring GBEVs, customizing GBEVs may be an effective strategy to accelerate their implementation and application. For example, through gene editing techniques, engineered bacteria could be used as “GBEV factories” that secrete GBEVs carrying specific genes, proteins, and polysaccharides, resulting in GBEVs with different bioactivities and targeting properties.^165^

At present, most studies related to GBEVs are based on experiments that use GBEVs isolated from *in vitro* cultures. However, GBEV composition is closely related to its growth environment. Whether the physiological characteristics of GBEVs secreted *in vitro* are the same as those of GBEVs secreted *in vivo* needs further investigation. In addition, studies on GBEVs have predominantly focused on the effects of GBEVs on the host, neglecting the effects on the intestinal microbiota. Although occasionally reported, the results are mostly invalid and suggestive.^166^ These unexplored mechanisms pertaining to the interactions between GBEVs and intestinal microbiota still needs to be supplemented with further knowledge. Detailed understanding of the interactions between GBEVs and intestinal microbiota will not only help to advance a better understanding of the interactions between bacteria in the intestine, but also help to better utilize GBEVs to regulate the intestinal microenvironment.

In conclusion, as presented in “125 Questions: Exploration and Discovery” published by “Science” journal,^167^ through questions such as “How is immune homeostasis maintained and regulated?” and “What role does our microbiome play in health and disease?” the role of GBEVs in the regulation of intestinal microenvironment requires further elucidation.

## Data Availability

Data sharing is not applicable to this article as no new data were created or analyzed in this review.
